# Supplementary material for: Characterization of the Key Aroma Compounds of Shandong Matcha Using HS-SPME-GC/MS and SAFE-GC/MS
Source: Foods. 2022 Sep 22;11(19):2964. doi: 10.3390/foods11192964 (PMC9562185; doi:10.3390/foods11192964)
Supplement: Supplementary file 1 [file foods-11-02964-s001.zip › foods-1891326-Supplementary.pdf]

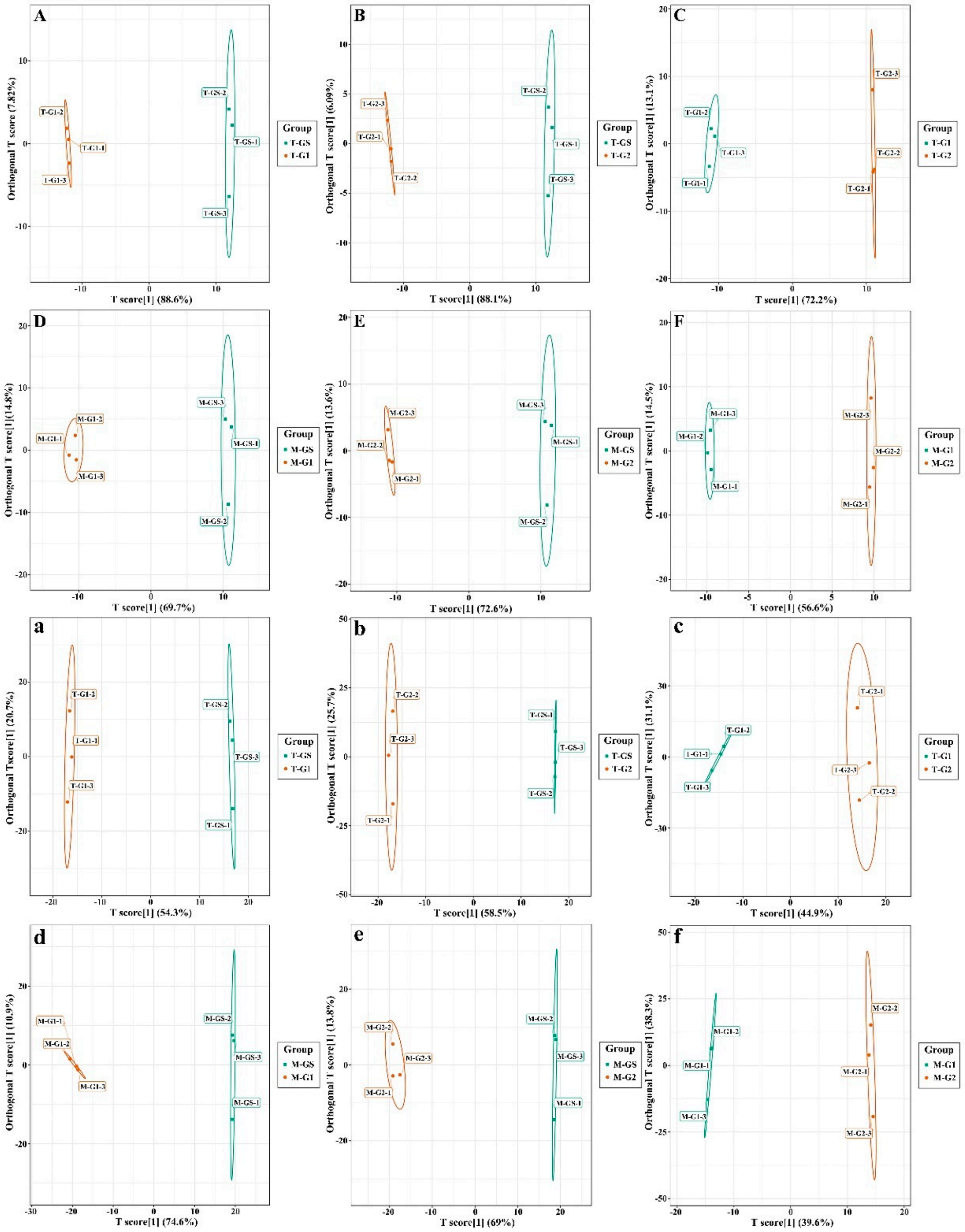

Figure S1. OPLS-DA analysis results of Shandong matcha and tencha. (**A-F**) HS-SPME.(**A**)T-GS and T-G1. (**B**) T-GS and T-G2. (**C**)T-G1 and T-G2. (**D**) M-GS and M-G1. (**E**) M-GS and M-G2. (**F**) M-G1 and M-G2. (**a-f**) SAFE. (**a**) T-GS and T-G1. (**b**) T-GS and T-G2. (**c**) T-G1 and T-G2. (**d**) M-GS and M-G1. (**e**) M-GS and M-G2. (**f**) M-G1 and M-G2.

Table S1. Comparison of volatile components extracted from Matcha and tencha by different methods.

Class	HS-SPME（Peak Area）						SAFE（Concentration）					
	M-GS	M-G1	M-G2	T-GS	T-G1	T-G2	M-GS	M-G1	M-G2	T-GS	T-G1	T-G2
Alcohol	2.62	2.21	2.25	2.93	2.56	1.65	7.69	4.97	4.57	5.64	4.22	3.68
Aromatics	3.47	3.49	4.53	2.48	4.68	4.13	6.71	6.59	6.68	6.43	7.20	6.57
Aldehyde	1.10	0.67	0.90	1.25	1.49	1.14	4.02	3.45	3.24	3.98	3.34	3.34
Acid	0.88	0.97	1.64	1.20	1.86	2.19	3.88	4.11	4.38	3.74	5.12	5.42
Terpenoids	16.92	7.44	11.76	18.52	11.47	10.11	25.09	10.76	12.82	22.05	14.34	14.63
Hydrocarbons	11.65	8.86	12.80	8.30	14.03	13.92	11.13	18.78	16.17	12.45	16.10	13.66
Ketone	4.57	1.90	2.47	4.89	2.84	2.10	10.25	6.48	6.97	8.40	6.79	6.60
Heterocyclic compound	49.53	65.35	45.84	51.90	46.54	44.01	12.12	17.02	17.07	17.65	17.83	17.56
Ester	6.91	7.34	15.27	6.78	12.23	18.25	12.96	24.73	24.99	15.88	21.82	25.34
Others	2.34	1.76	2.55	1.76	2.32	2.49	6.16	3.11	3.11	3.79	3.24	3.20

Table S2. Differential compounds of different grades of Shandong matcha.

Compounds	Class	Odour quality	Method	Type		
				M-G1_vs _M-GS	M-G2_vs_M-GS	M-G2_vs_M-G1
Octanal, 2-(phenylmethylene)-	Acid	floral	SAFE	up	-	-
Butanoic Acid	Acid	cheesy	SAFE	up	up	-
Nonanoic Acid	Acid	waxy,dirty,cheesy,dairy	SPME	down	-	up
6-Octenoic Acid, 3,7-dimethyl-	Acid	green、grassy、floral	SAFE	down	down	
8-Methyl-6-nonenoic Acid	Acid	-	SAFE	-	up	-
2-Propenoic Acid, 3-phenyl-	Acid	balsamic	SAFE	up	-	-
9,12,15-Octadecatrienoic Acid, (Z,Z,Z)-	Acid	fatty	SAFE	down	down	
Gamolenic Acid	Acid	-	SAFE	down	down	
2-Octenoic Acid	Acid	-	SAFE	up	up	down
Glycerin	Alcohol	-	SAFE	down	down	
Cyclobutaneethanol, 1-methyl-2-(1-methylethenyl)-, cis-	Alcohol	-	SAFE	up	up	down
2,4-Heptadien-1-ol, (E,E)-	Alcohol	fatty、green、fruity	SAFE	up	up	-
1-Hexanol, 3,5,5-trimethyl-	Alcohol	green	SAFE	down	down	
2-Penten-1-ol, (Z)-	Alcohol	green	SAFE	up	up	-
3-Hexen-1-ol, (Z)-	Alcohol	green	SAFE	up	up	-
1-Octen-3-ol	Alcohol	earthy	SAFE	up	-	-
Phenylethyl Alcohol	Alcohol	floral	SAFE	-	down	
1-Decanol	Alcohol	fatty	SAFE	down	down	
3,6-Nonadien-1-ol, (E,Z)-	Alcohol	green	SAFE	up	up	-
Batilol	Alcohol	-	SAFE	down	down	

Compounds	Class	Odour quality	Method	Type		
				M-G1_vs _M-GS	M-G2_vs_M-GS	M-G2_vs_M-G1
2-Nonen-1-ol	Alcohol	fatty	SAFE	down	down	
3,7-Octadiene-2,6-diol, 2,6-dimethyl-	Alcohol	-	SAFE	up	up	-
2-Decen-1-ol, (E)-	Alcohol	fatty	SAFE	down	down	
Cyclohexanol, 2,6-dimethyl-	Alcohol	-	SAFE	up	up	-
4-Hexen-1-ol, acetate	Alcohol	-	SAFE	up	up	-
2-Hexadecanol	Alcohol	-	SAFE	up	up	-
Benzenemethanol, .alpha.-methyl-.alpha.-(1-methyl-2-propenyl)-	Alcohol	-	HS-SPME	down	down	up
Benzenemethanol, .alpha.-2-propenyl-	Alcohol	-	SAFE	up	up	-
(2-Fluorophenyl) methanol, 2-methylpropyl ether	Alcohol	-		-	up	-
Ethanol, 2-(4-ethylphenoxy)-	Alcohol	-	SAFE	up	up	-
(E)-2,6-Dimethylocta-5,7-dien-2-ol	Alcohol	-	SAFE	up	-	-
3-Cyclohexene-1-carboxAldehyde, 4-(4-methyl-3-pentenyl)-	Aldehyde	citrus、floral、marine	SAFE	down	down	
Hexanal	Aldehyde	green	SAFE	up	up	-
2-Hexenal	Aldehyde	green	SAFE	-	down	
Heptanal	Aldehyde	green	HS-SPME	down	-	
2,4-Hexadienal, (E,E)-	Aldehyde	green	SAFE	up	up	-
BenzeneacetAldehyde	Aldehyde	green	HS-SPME	up	up	-
2,4-Octadienal, (E,E)-	Aldehyde	green	SAFE	up	up	-
2-Nonenal	Aldehyde	fatty、green	SAFE	-	up	-
BenzAldehyde, 4-ethyl-	Aldehyde	fruity	SAFE	up	up	-
1-Cyclohexene-1-carboxAldehyde, 4-(1-methylethenyl)-	Aldehyde	herbal	SAFE	up	up	down
4-Isopropylcyclohexa-1,3-dienecarbAldehyde	Aldehyde	fatty、spicy	SAFE	up	up	-

Compounds	Class	Odour quality	Method	Type		
				M-G1_vs _M-GS	M-G2_vs_M-GS	M-G2_vs_M-G1
Undecanal	Aldehyde	waxy、floral、green	SAFE	down	down	
Hexadecanal	Aldehyde	cardboard	SAFE	down	down	
2-FurAldehyde diethyl acetal	Aldehyde	fruity、earthy、mushroom	SAFE	-	up	-
BenzAldehyde, 2-methyl-	Aldehyde	cherry	SAFE	up	up	-
BenzAldehyde, 3-methyl-	Aldehyde	fruity	SAFE	up	up	-
4-MethoxycinnamAldehyde	Aldehyde	spicy、cinnamon、sweet	SAFE	down	down	
trans-4-MethoxycinnamAldehyde	Aldehyde	spicy、floral、sweet	SAFE	down	down	
CinnamAldehyde, .alpha.-pentyl-	Aldehyde	floral	SAFE	up	up	-
BenzAldehyde, 3-ethyl-	Aldehyde	-	SAFE	up	-	-
BenzAldehyde, 2,4-dimethyl-	Aldehyde	naphthyl、cherry	SAFE	up	up	-
2,5-FurandicarboxAldehyde	Aldehyde	-	SAFE	up	up	-
AcetAldehyde, (3,3-dimethylcyclohexylidene)-, (Z)-	Aldehyde	-	SAFE	up	up	-
BenzAldehyde, 2,4-dihydroxy-3,6-dimethyl-	Aldehyde	-	SAFE	up	up	-
BenzeneacetAldehyde, .alpha.-methyl-4-(2-methylpropyl)-	Aldehyde	-	SAFE	-	down	down
Benzamide	Amine	-	SAFE	down	down	
CyclohexanAmine, N-cyclohexyl-	Amine	-	SAFE	up	up	-
BenzenAmine, 2-methoxy-5-nitro-	Amine	-	SAFE	down	down	
Benzene, 1,1'-[oxybis(methylene)]bis-	Aromatics	earthy	SAFE	up	up	-
Benzene, 1,2,4,5-tetramethyl-	Aromatics	-	SAFE	down	down	
Benzene, (2-nitroethyl)-	Aromatics	flower、spice	SAFE	up	down	down

Compounds	Class	Odour quality	Method	Type		
				M-G1_vs _M-GS	M-G2_vs_M-GS	M-G2_vs_M-G1
Estragole	Aromatics	anisic	SAFE	down	down	
Naphthalene	Aromatics	pungent	HS-SPME	down	down	
1,2,3-trimethoxy-5-(2-propenyl)-Benzene	Aromatics	spicy、floral	SAFE	up	-	-
Benzene, 1,2,3-trimethoxy-5-(1-propenyl)-, (E)-	Aromatics	spicy、floral	SAFE	up	up	-
Naphthalene, 1,2,3,4-tetrahydro-	Aromatics	-	SAFE	down	down	
Isoelemicin	Aromatics	-	SAFE	-	up	-
p-Hydroxybiphenyl	Aromatics	-	SAFE	down	down	
Benzene, 1-ethyl-3-methyl-	Aromatics	-	SAFE	down	-	
Benzene, 1-ethyl-2-methyl-	Aromatics	-	HS-SPME	down	down	
TRANS-ANETHOLE	Aromatics	licorice 甘草	SAFE	down	down	
Benzene, 1,4-dichloro-	Aromatics	-	SAFE	up	up	-
Anisole	Aromatics	phenolic	SAFE	-	down	
Asarone	Aromatics	-	SAFE	down	down	
Mesitylene	Aromatics	-	HS-SPME	down	down	
p-Xylene	Aromatics	plastic, green, pungent	SAFE	up	up	-
Hydrazine, (4-methoxyphenyl)-	Aromatics	-	SAFE	down	down	
3,4-Dimethoxytoluene	Aromatics	-	SAFE	up	up	-
.alpha.-Methylstyrene	Aromatics	-	SAFE	down	-	
Benzene, 1-propynyl-	Aromatics	-	HS-SPME	-	down	
Benzene, pentamethyl-	Aromatics	-	HS-SPME	-	down	
Benzene, 1,2-diethyl-3,4-dimethyl-	Aromatics	-	SAFE	down	down	
1-(3-Methylbutyl)-2,3,5,6-tetramethylbenzene	Aromatics	-		-	-	down
(5-bromopentyl)-Benzene,	Aromatics	-	SAFE	up	up	-

Compounds	Class	Odour quality	Method	Type		
				M-G1_vs _M-GS	M-G2_vs_M-GS	M-G2_vs_M-G1
(3-Bromo-1-methylpropoxymethyl)benzene	Aromatics	-	SAFE	up	up	-
Benzene, 1,2-diethyl-3,4,5,6-tetramethyl-	Aromatics	-	SAFE	up	up	-
Benzene, 2-ethyl-1,4-dimethyl-	Aromatics	-	SAFE	up	up	-
Naphthalene, 1,2,3,4,4a,5,6,7-octahydro-4a-methyl-	Aromatics	-	SAFE	up	up	-
Naphthalene, 1,2-dihydro-4,5,7-trimethyl-	Aromatics	-	HS-SPME	-	down	
Propanoic acid, hexyl ester	Ester	fruity	SAFE	down	-	
n-Capric acid isobutyl ester	Ester	fermented、oily、sweet	SAFE	down	down	
3-Hexen-1-ol, formate, (Z)-	Ester	green	HS-SPME	down	down	
1,3-Cyclohexadiene-1-carboxylic acid, 2,6,6-trimethyl-, ethyl ester	Ester	floral	SAFE	up	up	-
2-Furancarboxylic acid, hexyl ester	Ester	floral	HS-SPME	down	down	
3-Hexen-1-ol, acetate, (Z)-	Ester	green	SAFE	down	down	down
Butanoic acid, 3-hexenyl ester, (Z)-	Ester	green	HS-SPME	down	down	down
cis-3-Hexenyl-.alpha.-methylbutyrate	Ester	green	HS-SPME	down	down	down
Methyl salicylate	Ester	minty	HS-SPME	down	down	
Isobornyl formate	Ester	balsamic	SAFE	up	up	-
Bicyclo[2.2.1]heptan-2-ol, 1,7,7-trimethyl-, formate, endo-	Ester	green、earthy、herbal	SAFE	up	up	-
trans-Geranic acid methyl ester	Ester	waxy	SAFE	down	down	
Acetic acid, nonyl ester	Ester	waxy	SAFE	down	down	
Hexanoic acid, 3-hexenyl ester, (Z)-	Ester	green	HS-SPME	down	down	down
Hexanoic acid, hexyl ester	Ester	green	HS-SPME	down	down	down
Butanoic acid, 3-methyl-, 1-ethenyl-1,5-dimethyl-4-hexenyl ester	Ester	herbal、cirtus	SAFE	up	-	down
Dodecanoic acid, methyl ester	Ester	waxy		-	-	down

Compounds	Class	Odour quality	Method	Type		
				M-G1_vs _M-GS	M-G2_vs_M-GS	M-G2_vs_M-G1
Butanoic acid, 3,7-dimethyl-2,6-octadienyl ester, (E)-	Ester	fruity	HS-SPME	down	down	
Benzoic acid, 1-methylethyl ester	Ester	floral	SAFE	up	up	-
.delta.-Dodecalactone	Ester	tropical	SAFE	down	-	
Hexanedioic acid, dimethyl ester	Ester	nutty	SAFE	up	up	-
Fenobucarb	Ester	-	HS-SPME	up	up	-
(S)-4-(1-Acetoxyallyl)phenyl acetate	Ester	-	SAFE	up	up	-
4-Pentenyl Acetate	Ester	green	SAFE	up	up	-
n-Caproic acid vinyl ester	Ester	-	SAFE	down	down	
2(4H)-Benzofuranone, 5,6,7,7a-tetrahydro-4,4,7a-trimethyl-, (R)-	Ester	musk	HS-SPME	up	up	-
E-2-Hexenyl benzoate	Ester	-	SAFE	down	down	down
Methylparaben	Ester	-	SAFE	up	up	-
4-Decenoic acid, methyl ester, Z-	Ester	fruity、fishy、green	SAFE	down	down	
9,12,15-Octadecatrienoic acid, methyl ester	Ester	oily、fatty、fruity	SAFE	down	down	
Benzoic acid, 4-ethoxy-, ethyl ester	Ester	-	SAFE	up	up	-
Isopropyl myristate	Ester	fatty	SAFE	-	down	down
n-Propyl acetate	Ester	fruity	SAFE	-	up	up
Hexadecanoic acid, methyl ester	Ester	waxy	HS-SPME	down	down	
1,2-Benzenedicarboxylic acid, bis(2-methylpropyl) ester	Ester	-	HS-SPME	down	-	
cis-3-Hexenyl cis-3-hexenoate	Ester	green	HS-SPME	down	down	down
3-Hexen-1-ol benzoate	Ester	-	HS-SPME	down	down	
Dehydromevalonic lactone	Ester	-	SAFE	up	up	-
9,12,15-Octadecatrienoic acid, methyl ester, (Z,Z,Z)-	Ester	-	HS-SPME	down	down	
Sulfurous acid, isobutyl pentyl ester	Ester	-	HS-SPME	down	down	

Compounds	Class	Odour quality	Method	Type		
				M-G1_vs _M-GS	M-G2_vs_M-GS	M-G2_vs_M-G1
Benzoic acid, 2-propenyl ester	Ester	fruity	SAFE	up	-	-
l-Alanine, N-(2,3,4-trifluorobenzoyl)-, methyl ester	Ester	-	HS-SPME	down	down	down
(1R,5S,6R)-2,7,7-Trimethylbicyclo[3.1.1]hept-2-en-6-yl acetate	Ester	-	SAFE	up	up	-
4-Methylhexyl 3-methylbutanoate	Ester	-	SAFE	down	down	
2-Butenoic acid, 2-methyl-, 2-methylpropyl ester	Ester	-	SAFE	up	up	-
Butanoic acid, cyclopentyl ester	Ester	-	SAFE	down	down	
2-Butenoic acid, 3-hexenyl ester, (E,Z)-	Ester	green	HS-SPME	down	down	
Hexanoic acid, 3-hexenyl ester	Ester	-	SAFE	-	down	down
Ethyl mandelate	Ester	-	SAFE	up	up	-
Butanoic acid, 2-methyl-, 3,7-dimethyl-2,6-octadienyl ester, (E)-	Ester	fruity	SAFE	up	-	-
Propanoic acid, 2-methyl-, butyl ester	Ester	fruity	SAFE	up	up	-
Propanoic acid, 2-methyl-, 2-methylbutyl ester	Ester	fruity	SAFE	down	-	
Phosphinic acid, diethyl-, methyl ester	Ester	-	HS-SPME	down	down	
Benzoic acid, 3,4-dimethyl-, methyl ester	Ester	-	SAFE	-	up	-
Ethyl tridecanoate	Ester	-	SAFE	up	-	-
Octanoic acid, 3-hexenyl ester, (Z)-	Ester	green	HS-SPME	down	down	
Pentadecanoic acid, 14-methyl-, methyl ester	Ester	-	SAFE	down	down	down
(6,6-dimethyl-2-bicyclo[3.1.1]hept-2-enyl)methyl 2-methylpropanoate	Ester	woody、fruity	SAFE	up	up	-
Geranyl isobutyrate	Ester	floral	SAFE	-	down	down
Sulfurous acid, hexyl pentadecyl ester	Ester	-	HS-SPME	down	-	up
Sulfurous acid, 2-ethylhexyl hexyl ester	Ester	-	HS-SPME	down	-	up
2-(2-butoxyethoxy)-Ethanol,acetate	Ester	-	HS-SPME	down	down	down
(Z,Z)-9,15-Octadecadienoic acid, methyl ester	Ester	-	HS-SPME	down	down	

Compounds	Class	Odour quality	Method	Type		
				M-G1_vs _M-GS	M-G2_vs_M-GS	M-G2_vs_M-G1
(E)-Hexanoic acid, 2-hexenyl ester	Ester	green		-	-	
Benzene, 1-methoxy-2-methyl-	Ether	sweet、nutty、floral	SAFE	down	down	down
Cyclopropanecarboxylic acid chloride	Halogenated hydrocarbons	-	SAFE	-	up	-
1-iodo-Decane	Halogenated hydrocarbons	-	SAFE	up	up	-
2-Acetyl-3-methylpyrazine	Heterocyclic compound	nutty	SAFE	down	down	down
2H-Pyran-2-one, tetrahydro-6-nonyl-	Heterocyclic compound	waxy	SAFE	down	down	
Pyrazine, (2-methylpropyl)-	Heterocyclic compound	green、vegetable、fruity	SAFE	up	up	-
5,6,7,8-Tetrahydroquinoxaline	Heterocyclic compound	nutty	SAFE	down	down	
Sesquirosefuran	Heterocyclic compound	-	SAFE	down	down	
2H-Pyran-3-ol, 6-ethenyltetrahydro-2,2,6-trimethyl-	Heterocyclic compound	floral	SAFE	up	-	down
Ethanone, 1-(2-furanyl)-	Heterocyclic compound	balsamic	SAFE	down	down	
Furan, 2-pentyl-	Heterocyclic compound	fruity、green、earthy	SAFE	up	up	-

Compounds	Class	Odour quality	Method	Type		
				M-G1_vs _M-GS	M-G2_vs_M-GS	M-G2_vs_M-G1
Ethanone, 1-(1H-pyrrol-2-yl)-	Heterocyclic compound	musty	SAFE	-	down	down
Pyrazine, 2-ethyl-3-methyl-	Heterocyclic compound	nutt	SAFE	down	down	
Pyrazine, 3-ethyl-2,5-dimethyl-	Heterocyclic compound	nutty	SAFE	down	down	
Pyrazine, 2-ethyl-3,5-dimethyl-	Heterocyclic compound	nutty	HS-SPME	down	down	
2,3-Dimethyl-5-ethylpyrazine	Heterocyclic compound	roasted	SAFE	down	down	
rose furan epoxide	Heterocyclic compound	green	SAFE	up	up	down
Ethanethioic acid, S-(2-furanylmethyl) ester	Heterocyclic compound	sulfurous、roasted	SAFE	up	up	-
Indole	Heterocyclic compound	animal	HS-SPME	down	down	
Coumarin	Heterocyclic compound	tonka	HS-SPME	down	down	
2H-Pyran-2-one, 5,6-dihydro-6-pentyl-	Heterocyclic compound	coconut、sweet	SAFE	up	up	-
Hydrocoumarin	Heterocyclic compound	tonka	SAFE	up	up	-
Precocene I	Heterocyclic compound	-	HS-SPME	up	-	-

Compounds	Class	Odour quality	Method	Type		
				M-G1_vs _M-GS	M-G2_vs_M-GS	M-G2_vs_M-G1
2H-Indol-2-one, 1,3-dihydro-	Heterocyclic compound	-	SAFE	up	up	-
5,6-Dihydro-5-methyluracil	Heterocyclic compound	-	SAFE	up	up	-
Pentylenetetrazol	Heterocyclic compound	-	SAFE	down	down	
GrAmine	Heterocyclic compound	-	SAFE	up	up	-
Debrisoquine	Heterocyclic compound	-	SAFE	up	up	-
Pentobarbital	Heterocyclic compound	-	SAFE	down	-	
2-methyl-4-Quinolinol	Heterocyclic compound	-	SAFE	up	up	up
[1,3]Dioxolo[4,5-j]phenanthridine	Heterocyclic compound	-	SAFE	down	down	
Nifedipine	Heterocyclic compound	-	SAFE	down	down	
2(5H)-Furanone	Heterocyclic compound	buttery	SAFE	-	down	
Caffeine	Heterocyclic compound	-	HS-SPME	down	-	up
7,9-Di-tert-butyl-1-oxaspiro(4,5)deca-6,9-diene-2,8-dione	Heterocyclic compound	-	HS-SPME	down	-	

Compounds	Class	Odour quality	Method	Type		
				M-G1_vs _M-GS	M-G2_vs_M-GS	M-G2_vs_M-G1
1H-Pyrrole-3-carbonitrile	Heterocyclic compound	-	SAFE	up	up	-
2,2'-Ethylidenebis(5-methylfuran)	Heterocyclic compound	-	SAFE	up	-	-
1H-Pyrazolo[3,4-d]pyrimidin-4-amine	Heterocyclic compound	-	SAFE	up	-	-
1,4 Benzodioxan-6-amine	Heterocyclic compound	-	SAFE	up	up	-
2-Ethylpiperidine	Heterocyclic compound	-	SAFE	down	-	
Piperidin-3-one, 2,2,5-methyl-	Heterocyclic compound	-	SAFE	up	up	-
1-Pentanone, 1-(2-furanyl)-	Heterocyclic compound	sweet、caramellic	SAFE	up	up	-
6-Quinolinamine, 2-methyl-	Heterocyclic compound	-	SAFE	up	-	down
2-Piperidinimine	Heterocyclic compound	-	SAFE	up	-	-
Furan, 2-(1-pentenyl)-, (E)-	Heterocyclic compound	roasted	SAFE	up	up	-
1,2,4,5-Tetrazin-3-Amine	Heterocyclic compound	-	SAFE	down	-	
1,2,4,5-Tetrazine-3,6-diAmine	Heterocyclic compound	-	SAFE	up	up	-

Compounds	Class	Odour quality	Method	Type		
				M-G1_vs _M-GS	M-G2_vs_M-GS	M-G2_vs_M-G1
(E)-linalool oxide (pyranoid)	Heterocyclic compound	woody	HS-SPME	down	down	down
3-Methyl-4,5-diamino-1,2,4(4H)-triazole	Heterocyclic compound	-	SAFE	down	-	
4H-Pyran-4-one, 2,3-dihydro-3,5-dihydroxy-6-methyl-	Heterocyclic compound	-	SAFE	-	down	
1H-Pyrazole-1-carboximidamide, 3,5-dimethyl-	Heterocyclic compound	-	SAFE	up	up	-
3-Methylbenzothiophene	Heterocyclic compound	-	SAFE	down	down	
(5R,8aR)-5-Propyloctahydroindolizine	Heterocyclic compound	-	HS-SPME	down	-	
1H-Imidazole, 2-propyl-	Heterocyclic compound	-	SAFE	up	up	-
3,5-Dimethyl-1-butylpyrazole	Heterocyclic compound	-	SAFE	up	up	-
1,7-Diazabicyclo[2.2.0]heptane	Heterocyclic compound	-	SAFE	up	up	-
2-Thiophenecarboxaldehyde, 5-chloro-	Heterocyclic compound	-	SAFE	-	down	
Ethyl 2-(5-methyl-5-vinyltetrahydrofuran-2-yl)propan-2-yl carbonate	Heterocyclic compound	-	HS-SPME	down	down	
2-Hydroxymethylbenzimidazole	Heterocyclic compound	-	HS-SPME	up	-	-

Compounds	Class	Odour quality	Method	Type		
				M-G1_vs _M-GS	M-G2_vs_M-GS	M-G2_vs_M-G1
1-Pentanone, 1-(2-thienyl)-	Heterocyclic compound	-	SAFE	down	down	
4,4'-Bitriazolyl	Heterocyclic compound	-	SAFE	up	up	-
1,4-Methano-1H-Cyclopropa[d]pyridazine, 4,4a,5,5a-tetrahydro-6,6-dimethyl-, (1.alpha.,4.alpha.,4a.alpha.,5a.alpha.)-	Heterocyclic compound	-	SAFE	up	up	-
2,5-Furandione, 3-methyl-4-propyl-	Heterocyclic compound	-	SAFE	down	down	
5-Isopropyl-3,3-dimethyl-2-methylene-2,3-dihydrofuran	Heterocyclic compound	-	SAFE	up	up	-
1H-Pyrrole-2,5-dione, 3-ethenyl-4-methyl-	Heterocyclic compound	-	SAFE	up	up	-
1H-Imidazole-4-methanol	Heterocyclic compound	-	SAFE	-	up	-
Cobalt, bis(.eta.-5-piperidinylcyclopentadienyl)-	Heterocyclic compound	-	HS-SPME	down	-	
2,3,5-Trimethyl-6-ethylpyrazine	Heterocyclic compound	-	SAFE	down	down	down
N-Benzyloxy-2-carbomethoxyaziridine	Heterocyclic compound	-	SAFE	up	up	-
Isomaltol	Heterocyclic compound	burnt、caramellic、fruity	SAFE	up	-	-
(2R,4aS,5S,8aS)-2,5-Dipentyldecahydroquinoline	Heterocyclic compound	-	SAFE	down	down	

Compounds	Class	Odour quality	Method	Type		
				M-G1_vs _M-GS	M-G2_vs_M-GS	M-G2_vs_M-G1
3-Formyl-4,5-dimethyl-pyrrole	Heterocyclic compound	-	HS-SPME	down	down	
diethylboryl-.delta.-Valerolactam	Heterocyclic compound	-	HS-SPME	up	up	-
2-Thiopheneacetic acid, but-3-yn-2-yl ester	Heterocyclic compound	-	HS-SPME	-	down	
(5S,8R,8aS)-8-Methyl-5-((Z)-pent-2-en-4-yn-1-yl)octahydroindolizine	Heterocyclic compound	-	HS-SPME	-	down	
6-methyl-3(2H)-Pyridazinone	Heterocyclic compound	-	HS-SPME	down	down	
4,6-dimethyl-2-Pyridinemethanol,acetate (ester)	Heterocyclic compound	-	HS-SPME	down	down	
2,3-dihydro-4H-1-Benzopyran-4-one	Heterocyclic compound	-	HS-SPME	down	down	
2-Formyl-4,5-dimethyl-pyrrole	Heterocyclic compound	-	HS-SPME	down	down	
Adamantane	Hydrocarbons	-	SAFE	down	down	
Octane	Hydrocarbons	gasoline	SAFE	up	up	-
Nonane	Hydrocarbons	gasoline	SAFE	up	up	-
Decane	Hydrocarbons	-	HS-SPME	down	-	
Dodecane	Hydrocarbons	-	HS-SPME	down	-	
Tetradecane	Hydrocarbons	-	HS-SPME	down	-	
4-methyl-1-(1-methylethyl)-Bicyclo[3.1.0]hex-2-ene	Hydrocarbons	-	HS-SPME	-	up	-
1-Nonene, 4,6,8-trimethyl-	Hydrocarbons	-	SAFE	up	up	-

Compounds	Class	Odour quality	Method	Type		
				M-G1_vs _M-GS	M-G2_vs_M-GS	M-G2_vs_M-G1
Heptadecane, 2-methyl-	Hydrocarbons	-	SAFE	down	down	
Tetradecane, 2,6,10-trimethyl-	Hydrocarbons	-	SAFE	down	down	
E-1-Methoxy-4-hexene	Hydrocarbons	-	SAFE	up	up	-
Cyclohexene, 4-methyl-1-(1-methylethenyl)-	Hydrocarbons	-	SAFE	up	up	-
Undecane, 3,6-dimethyl-	Hydrocarbons	-	HS-SPME	-	down	
Albene	Hydrocarbons	-	SAFE	up	up	-
1-Hexen, 2-(p-anisyl)-5-methyl-	Hydrocarbons	-	HS-SPME	up	up	-
1,5-Heptadiene, 2,6-dimethyl-	Hydrocarbons	-	SAFE	-	down	
Octane, 2,6,6-trimethyl-	Hydrocarbons	-	HS-SPME	down	down	
Nonane, 3-methyl-5-propyl-	Hydrocarbons	-	SAFE	up	up	-
3-ethyl-Undecane	Hydrocarbons	-	HS-SPME	-	up	-
2-butyl-1,3,3-trimethyl-Cyclohexene	Hydrocarbons	-	HS-SPME	-	down	
Hexacosane	Hydrocarbons	-	SAFE	down	down	
3-Hexanone, 1-phenyl-	Ketone	-	SAFE	up	up	-
Cyclopentanone, 2-(2-hexenyl)-	Ketone	floral	SAFE	down	down	
4-Acetylanisole	Ketone	anisic	HS-SPME	up	up	-
2-methyl-3-Pentanone	Ketone	mint	SAFE	down	down	
2-Heptanone	Ketone	cheesy		-	-	down
5-Hepten-2-one, 6-methyl-	Ketone	citrus	SAFE	up	-	-
(5Z)-Octa-1,5-dien-3-one	Ketone	green	HS-SPME	down	-	up
3,5-Octadien-2-one	Ketone	fatty、fruity、mushroom	HS-SPME	up	up	-
2-Cyclohexen-1-one, 4-(3-hydroxy-1-butenyl)-3,5,5-trimethyl-	Ketone	spicy	SAFE	up	up	-

Compounds	Class	Odour quality	Method	Type		
				M-G1_vs _M-GS	M-G2_vs_M-GS	M-G2_vs_M-G1
1,2-Propanedione, 1-phenyl-	Ketone	plastic、buttery、honey	SAFE	up	up	-
2-Butanone, 4-(4-hydroxy-3-methoxyphenyl)-	Ketone	spicy	SAFE	up	up	-
Methanone, (4-hydroxyphenyl)phenyl-	Ketone	-	SAFE	up	up	-
Isophorone	Ketone	woody	SAFE	up	up	-
Acetophenone, 4'-hydroxy-	Ketone	floral	SAFE	up	up	-
Ethanone, 1-(2-methyl-1-cyclopenten-1-yl)-	Ketone	-	SAFE	up	up	-
3,5-Octadien-2-one, (E,E)-	Ketone	fruity、green、grassy	SAFE	up	up	-
1H-Pyrrole-2,5-dione, 3-ethyl-4-methyl-	Ketone	-	HS-SPME	up	up	-
2-Cyclopenten-1-one, 3-methyl-2-(2-pentenyl)-, (Z)-	Ketone	floral	HS-SPME	down	down	
Cyclohexanone, 2,2,6-trimethyl-	Ketone	thujonic、pungent	SAFE	up	up	-
Ethanone, 1-(3-hydroxyphenyl)-	Ketone	-	SAFE	up	up	-
2-Cyclohexen-1-one, 3-methyl-	Ketone	nutty	SAFE	up	up	-
1-Butanone, 2-hydroxy-1-phenyl-	Ketone	-	SAFE	up	-	-
Ethanone, 1-(1H-pyrazol-4-yl)-	Ketone	-	HS-SPME	up	up	-
Ethanone, 1-(2,4-dimethylphenyl)-	Ketone	floral	SAFE	up	up	-
2,6,6-Trimethyl-2-cyclohexene-1,4-dione	Ketone	musty、woody、sweet、tea	SAFE	up	up	-
2-Buten-1-one, 1-(2,6,6-trimethyl-1-cyclohexen-1-yl)-	Ketone	fruity	HS-SPME	up	up	-
Ethanone, 1-(1,3-dimethyl-3-cyclohexen-1-yl)-	Ketone	-	SAFE	up	up	-
2(1H)-Naphthalenone, 3,4,5,6,7,8-hexahydro-	Ketone	-	SAFE	up	up	-
4-Isopropyl-1,3-cyclohexanedione	Ketone	-	SAFE	-	down	
2-Pentadecanone	Ketone	floral	SAFE	down	down	

Compounds	Class	Odour quality	Method	Type		
				M-G1_vs _M-GS	M-G2_vs_M-GS	M-G2_vs_M-G1
4,6-Octadiyn-3-one, 2-methyl-	Ketone	-	HS-SPME	down	down	
2-Cyclohexen-1-one, 4-hydroxy-3-methyl-6-(1-methylethyl)-, trans-	Ketone	-	SAFE	-	down	
4-(2,6,6-Trimethylcyclohexa-1,3-dienyl)but-3-en-2-one	Ketone	-	HS-SPME	up	up	-
1-(4-tert-Butylphenyl)propan-2-one	Ketone	-	HS-SPME	up	up	-
5-methyl-1,2,5,6-Tetrahydropyridin-2-one	Nitrogen compounds	-	HS-SPME	down	down	
1-isocyano-3-methyl-Benzene	Nitrogen compounds	-	HS-SPME	down	down	down
Butanoic acid, anhydride	Others	butter	SAFE	up	up	-
Hydroquinone	Phenol	-	SAFE	-	up	-
2-methoxy-Phenol	Phenol	phenolic	SAFE	up	up	-
p-Cresol	Phenol	phenolic	SAFE	down	down	
Phenol, 3-methyl-	Phenol	phenolic	SAFE	down	down	
Phenol, 4-propyl-	Phenol	phenolic、medicinal	SAFE	up	up	-
Phenol, 2-(1,1-dimethylethyl)-	Phenol	-	SAFE	up	up	-
Phenol, 4,4'-methylenebis-	Phenol	-	SAFE	down	down	
Phenol, 2-(1-methylpropyl)-	Phenol	-	SAFE	up	up	-
1H-Inden-5-ol, 2,3-dihydro-	Phenol	-	HS-SPME	down	down	down
8,9-Dehydrothymol	Phenol	-	SAFE	down	down	
Phenol, 2,4,6-tris(1-methylethyl)-	Phenol	-	HS-SPME	-	down	
Diallyl disulphide	Sulfur compounds	alliaceous、onion	SAFE	up	-	-

Compounds	Class	Odour quality	Method	Type		
				M-G1_vs _M-GS	M-G2_vs_M-GS	M-G2_vs_M-G1
Furan, 2-[(methyldithio)methyl]-	Sulfur compounds	alliaceous、sulfurous、roasted	SAFE	up	up	-
3-Cyclohexene-1-methanethiol, .alpha.,.alpha.,4-trimethyl-	Sulfur compounds	citrus	SAFE	up	up	-
Thiocyanic acid, phenylmethyl ester	Sulfur compounds	-	SAFE	-	up	-
Benzene, (isothiocyanatomethyl)-	Sulfur compounds	watercress	SAFE	up	up	-
TriSulfur compounds, dipropyl	Sulfur compounds	sulfurous、alliaceous	SAFE	down	down	
2-Methylthiolane, S,S-dioxide	Sulfur compounds	-	HS-SPME	down	down	down
(1aR,1bS,2aS,5S,5aS,7aS)-2,2,5,7a-Tetramethyldecahydrocyclopenta[2',3']cyclobuta[1',2':3,4]benzo[1,2-b]oxirene	Terpenoids	-	SAFE	up	up	-
Cyclohexene, 4-[(1E)-1,5-dimethyl-1,4-hexadien-1-yl]-1-methyl-	Terpenoids	-	SAFE	down	down	down
.beta.-Acorenol	Terpenoids	-	SAFE	up	up	-
(5R,10R)-10-Methyl-6-methylene-2-(propan-2-ylidene)spiro[4.5]dec-7-ene	Terpenoids	-	SAFE	up	up	-
Naphthalene, decahydro-1,6-bis(methylene)-4-(1-methylethyl)-, (4.alpha.,4a.alpha.,8a.alpha.)-	Terpenoids	-	SAFE	-	down	down
Kessane	Terpenoids	-	SAFE	down	down	
Carvone oxide, trans-	Terpenoids	-	SAFE	up	up	-
.alpha.-Pinene	Terpenoids	herbal	SAFE	up	up	-
3-Carene	Terpenoids	citrus	SAFE	-	up	up
Linalool	Terpenoids	floral	SAFE	down	down	
Bicyclo[3.1.0]hexan-2-ol, 2-methyl-5-(1-methylethyl)-, (1.alpha.,2.alpha.,5.alpha.)-	Terpenoids	woody、balsam	SAFE	up	up	-

Compounds	Class	Odour quality	Method	Type		
				M-G1_vs _M-GS	M-G2_vs_M-GS	M-G2_vs_M-G1
2-Methylisoborneol	Terpenoids	earthy、musty	HS-SPME	down	down	
4-Hexen-1-ol, 5-methyl-2-(1-methylethenyl)-, (R)-	Terpenoids	herbal	SAFE	up	up	-
Carveol	Terpenoids	minty	SAFE	-	down	down
2,6-Octadienal, 3,7-dimethyl-, (Z)-	Terpenoids	citrus	SAFE	up	up	-
4-(1-methylethyl)-Benzaldehyde	Terpenoids	spicy	SAFE	down	down	
safranal	Terpenoids	herbal	SAFE	up	up	down
(-)-.beta.-Bourbonene	Terpenoids	herbal、woody、floral	SAFE	-	down	
.alpha.-Ionone	Terpenoids	floral	HS-SPME	up	up	-
(E)-.beta.-Famesene	Terpenoids	woody、citrus、herbal	HS-SPME	down	down	down
trans-.beta.-Ionone	Terpenoids	floral	HS-SPME	-	up	-
Humulene	Terpenoids	woody	SAFE	up	up	-
1,3-Cyclohexadiene, 5-(1,5-dimethyl-4-hexenyl)-2-methyl-, [S-(R*,S*)]-	Terpenoids	spicy fresh sharp	SAFE	up	up	-
.gamma.-Muurolene	Terpenoids	woody	SAFE	up	-	-
(1S,2E,6E,10R)-3,7,11,11-Tetramethylbicyclo[8.1.0]undeca-2,6-diene	Terpenoids	green、woody、weedy	SAFE	up	up	-
.alpha.-Farnesene	Terpenoids	woody	HS-SPME	down	down	down
Cyclohexene, 3-(1,5-dimethyl-4-hexenyl)-6-methylene-, [S-(R*,S*)]-	Terpenoids	herbal、fruity、woody	SAFE	down	down	
(E)-nerolidol<br>(E)-linalool oxide (pyranoid)	Terpenoids	floral	HS-SPME	down	down	
3-Buten-2-one, 4-(2,2,6-trimethyl-7-oxabicyclo[4.1.0]hept-1-yl)-	Terpenoids	fruity	HS-SPME	up	up	-

Compounds	Class	Odour quality	Method	Type		
				M-G1_vs _M-GS	M-G2_vs_M-GS	M-G2_vs_M-G1
.alpha.-Cadinol	Terpenoids	woody, herbal	SAFE	up	up	-
10-epi-.gamma.-Eudesmol	Terpenoids	woody	SAFE	up	up	-
Fenchone	Terpenoids	-	SAFE	up	up	-
(E)-4,8-Dimethylnona-1,3,7-triene	Terpenoids	-	SAFE	up	-	down
L-.alpha.-Terpineol	Terpenoids	terpenic、lilac、floral	HS-SPME	-	down	
(+)-neodihydrocarveol	Terpenoids	-	SAFE	up	up	-
D-Verbenone	Terpenoids	-	SAFE	up	up	-
2,6-Octadien-1-ol, 3,7-dimethyl-	Terpenoids	-	SAFE	down	-	
2-Cyclohexen-1-one, 2-hydroxy-3-methyl-6-(1-methylethyl)-	Terpenoids	minty	SAFE	up	up	-
Furan, 3-(4,8-dimethyl-3,7-nonadienyl)-, (E)-	Terpenoids	-	SAFE	up	up	-
3-Hydroxy-7,8-dihydro-.beta.-ionol	Terpenoids	-	SAFE	up	up	-
Eremophilene	Terpenoids	-	SAFE	up	up	-
Pinocarvone	Terpenoids	minty	SAFE	down	-	
beta-Elemen	Terpenoids	sweet	SAFE	up	up	-
Pinocamphone	Terpenoids	spicy	SAFE	up	up	-
(+)-alpha-Pinene	Terpenoids	herbal	SAFE	up	up	-
d-Nerolidol	Terpenoids	floral	SAFE	down	-	
Benzene, 1-(1,5-dimethylhexyl)-4-methyl-	Terpenoids	-	HS-SPME	-	down	
(+)-3-Carene	Terpenoids	sweet	SAFE	-	up	up
1,3,6,10-Cyclotetradecatetraene, 3,7,11-trimethyl-14-(1-methylethyl)-, [S-(E,Z,E,E)]-	Terpenoids	-	SAFE	-	up	-
(1R,4R,4aS,8aR)-4,7-Dimethyl-1-(prop-1-en-2-yl)-1,2,3,4,4a,5,6,8a-octahydronaphthalene	Terpenoids	-	SAFE	up	-	down

Compounds	Class	Odour quality	Method	Type		
				M-G1_vs _M-GS	M-G2_vs_M-GS	M-G2_vs_M-G1
(2R,3R,6S)-6-Isopropyl-3-methyl-2-(prop-1-en-2-yl)-3-vinylcyclohexanone	Terpenoids	-	SAFE	up	up	-
[1α,4aα,8aα]-1,2,4a,5,6,8a-hexahydro-4-7-dimethyl-1-[1-methylethyl]naphthalene	Terpenoids	-	SAFE	up	up	-
(1S,4S,4aS)-1-Isopropyl-4,7-dimethyl-1,2,3,4,4a,5-hexahydronaphthalene	Terpenoids	-	SAFE	up	down	down
Benzene, 1-methyl-4-(1,2,2-trimethylcyclopentyl)-, (R)-	Terpenoids	-	SAFE	down	down	
1,1,7,7a-Tetramethyl-1a,2,6,7,7a,7b-hexahydro-1H-cyclopropa[a]naphthalene	Terpenoids	-	SAFE	up	up	-
.alpha.-Corocalene	Terpenoids	-	SAFE	-	up	-
Bicyclo[3.1.1]hept-2-ene, 2,6-dimethyl-6-(4-methyl-3-pentenyl)-	Terpenoids	-	SAFE	up	up	-
Bicyclo[2.2.1]heptane, 2-chloro-1,7,7-trimethyl-, (1R-endo)-	Terpenoids	-	SAFE	up	up	-
Bicyclo[3.1.1]hept-3-en-2-one, 4,6,6-trimethyl-, (1S)-	Terpenoids	spicy、camphoreous	SAFE	up	-	-
1-Phenanthrenecarboxaldehyde, 1,2,3,4,4a,9,10,10a-octahydro-1,4a-dimethyl-7-(1-methylethyl)-, [1R-(1.alpha.,4a.beta.,10a.alpha.)]-	Terpenoids	-	SAFE	down	down	
(-)-Aristolene	Terpenoids	-	SAFE	up	-	down
Palustrol (Ledum)	Terpenoids	-	SAFE	down	down	
Bicyclo[3.1.0]hex-2-ene, 4-methylene-1-(1-methylethyl)-	Terpenoids	-	SAFE	down	-	
Valerena-4,7(11)-diene	Terpenoids	-	SAFE	-	down	down
Cyclopropanecarboxylic Acid, 2,2-dimethyl-3-(2-methyl-1-propenyl)-, (1R-trans)-	Terpenoids	-	SAFE	up	up	-

Compounds	Class	Odour quality	Method	Type		
				M-G1_vs _M-GS	M-G2_vs_M-GS	M-G2_vs_M-G1
1(2H)-Naphthalenone, 3,4,4a,5,6,7-hexahydro-4a,5-dimethyl-3-(1-methylethenyl)-, [3S-(3.alpha.,4a.alpha.,5.alpha.)]-	Terpenoids	-	SAFE	up	up	-
Longifolenaldehyde	Terpenoids	-	SAFE	up	up	-
Tricyclo[2.2.1.0(2,6)]heptane-3-methanol, 2,3-dimethyl-	Terpenoids	-	SAFE	up	up	-
1-Isopropyl-4,7-dimethyl-1,2,3,5,6,8a-hexahydronaphthalene	Terpenoids	-	SAFE	up	up	down
Carvenone	Terpenoids	minty	SAFE	up	up	-
(1R,5R)-2-Methyl-5-((R)-6-methylhept-5-en-2-yl)bicyclo[3.1.0]hex-2-ene	Terpenoids	-	HS-SPME	down	down	down
(R)-5,7-dimethyl-1,6-Octadiene	Terpenoids	-	HS-SPME	-	down	-

Table S3. Different aroma components of tencha ground into matcha.

Compounds	Class	Odour quality	Type		
			T-GS_vs_M-GS	T-G1_vs_M-G1	T-G2_vs_M-G2
CyclohexanAmine, N-cyclohexyl-	Amine	-	up	-	-
BenzenAmine, 2-methoxy-5-nitro-	Amine	-	down	-	-
5H-Tetrazol-5-Amine	Amine	-	-	down	-
Formamide, N-phenyl-	Amine	-	up	up	-
1,6-HexanediAmine, N,N,N',N'-tetramethyl-	Amine	-	-	up	up
Glycerin	Alcohol	-	down	up	up
Ethanol, 2-(3,3-dimethylcyclohexylidene)-, (Z)-	Alcohol	-	-	up	up
Cyclobutaneethanol, 1-methyl-2-(1-methylethenyl)-, cis-	Alcohol	-	up	-	-
2,4-Heptadien-1-ol, (E,E)-	Alcohol	fatty、green、fruity	-	down	down
1-Hexanol, 3,5,5-trimethyl-	Alcohol	green	down	down	down
(S)-2,5-Dimethyl-3-vinylhex-4-en-2-ol	Alcohol	-	up	-	-
1-Pentanol	Alcohol	fermented、oily、sweet	-	down	-
3-Hexen-1-ol, (Z)-	Alcohol	green	up	-	-
2-Octen-1-ol, (E)-	Alcohol	green	-	up	up
1-Decanol	Alcohol	fatty	down	up	up
3,6-Nonadien-1-ol, (E,Z)-	Alcohol	green	up	-	-
1,2-Octanediol	Alcohol	-	-	up	up
Batilol	Alcohol	-	down	-	-
Ethanol, 2-butoxy-	Alcohol	-	-	up	up
3,7-Octadiene-2,6-diol, 2,6-dimethyl-	Alcohol	-	up	down	-
6-Nonen-1-ol, (E)-	Alcohol	-	-	up	up
2-Decen-1-ol, (E)-	Alcohol	fatty	-	up	up
Cyclohexanemethanol, .alpha.,.alpha.-dimethyl-4-methylene-	Alcohol	-	up	up	up

Compounds	Class	Odour quality	Type		
			T-GS_vs_M-GS	T-G1_vs_M-G1	T-G2_vs_M-G2
Cyclohexanol, 1-ethenyl-	Alcohol	-	up	up	up
6-Undecanol	Alcohol	-	up	-	-
4-Hexen-1-ol, acetate	Alcohol	-	up	up	up
Benzenemethanol, .alpha.-methyl-.alpha.-(1-methyl-2-propenyl)-	Alcohol	-	up	up	up
(2,2,6-Trimethyl-bicyclo[4.1.0]hept-1-yl)-methanol	Alcohol	-	-	up	up
3,7,11,15-tetramethyl-1-Hexadecyn-3-ol	Alcohol	-	-	up	-
Benzene, (2,2-dimethoxyethyl)-	Aromatics	green	up	up	up
Benzene, 1,2,4-trimethyl-	Aromatics	-	-	up	up
Cyclohexene, 1-methoxy-	Aromatics	herbal	-	up	up
Benzene, 1,2,4,5-tetramethyl-	Aromatics	sweet	down	-	-
Benzene, (2-nitroethyl)-	Aromatics	flower、spice	up	-	-
Estragole	Aromatics	anisic	down	-	-
Naphthalene	Aromatics	pungent	down	down	down
Naphthalene, 1,2,3,4-tetrahydro-	Aromatics	-	down	-	-
Triphenylene	Aromatics	-	up	up	up
Benzene, 1-ethyl-3-methyl-	Aromatics	-	-	up	up
Benzene, 1-ethyl-2-methyl-	Aromatics	-	down	-	-
Naphthalene, 1-methyl-	Aromatics	naphthyl、camphoreous	-	up	up
TRANS-ANETHOLE	Aromatics	licorice	down	-	-
Benzene, 1,2-dichloro-	Aromatics	-	-	down	down
Benzene, 1,4-dichloro-	Aromatics	-	up	-	-
Asarone	Aromatics	-	down	-	-
Mesitylene	Aromatics	-	down	down	down

Compounds	Class	Odour quality	Type		
			T-GS_vs_M-GS	T-G1_vs_M-G1	T-G2_vs_M-G2
p-Xylene	Aromatics	Plastic, green, pungent	up	-	-
Hydrazine, (4-methoxyphenyl)-	Aromatics	-	down	-	-
.alpha.-Methylstyrene	Aromatics	-	up	up	up
Naphthalene, 2-methyl-	Aromatics	floral	-	-	down
Benzene, pentamethyl-	Aromatics	-	-	down	down
Benzene, 1,2-diethyl-3,4-dimethyl-	Aromatics	-	down	-	-
(5-bromopentyl)-Benzene,	Aromatics	-	up	-	-
Benzene, 2-ethyl-1,4-dimethyl-	Aromatics	-	up	-	-
Naphthalene, 1,2,3,4,4a,5,6,7-octahydro-4a-methyl-	Aromatics	-	up	-	up
Naphthalene, 1,2-dihydro-4,5,7-trimethyl-	Aromatics	-	-	-	down
Hydroquinone	Phenol	-	-	up	up
p-Cresol	Phenol	phenolic	up	up	up
Phenol, 3-methyl-	Phenol	phenolic	-	up	up
Phenol, 4-propyl-	Phenol	phenolic、medicinal	up	-	-
Phenol, 4-(1-methyl-1-phenylethyl)-	Phenol	-	-	-	up
Phenol, 4,4'-methylenebis-	Phenol	-	down	-	-
1H-Inden-5-ol, 2,3-dihydro-	Phenol	-	down	-	-
Phenol, 2,5-bis(1-methylpropyl)-	Phenol	-	-	down	-
8,9-Dehydrothymol	Phenol	-	down	-	-
Phenol, 2,4,6-tris(1-methylethyl)-	Phenol	-	-	down	-
2-(1,1-Dimethylethyl)-6-(1-methylethyl)phenol	Phenol	-	-	down	-
Tricyclo[3.3.1.1(3,7)]decane, 2-nitro-	Nitrogen compounds	-	-	up	up
5-methyl-1,2,5,6-Tetrahydropyridin-2-one	Nitrogen compounds	-	-	up	-

Compounds	Class	Odour quality	Type		
			T-GS_vs_M-GS	T-G1_vs_M-G1	T-G2_vs_M-G2
3-Cyclohexene-1-methanethiol, .alpha.,.alpha.,4-trimethyl-	Sulfur compounds	citrus	-	-	up
TriSulfur compounds, dipropyl	Sulfur compounds	sulfurous	down	-	-
2-Methylthiolane, S,S-dioxide	Sulfur compounds	-	-	-	down
1-iodo-Decane	Halogenated hydrocarbons	-	up	down	-
Dimethylphosphinic fluoride	Halogenated hydrocarbons	-	-	up	-
1-iodo-Hexadecane	Halogenated hydrocarbons	-	up	-	-
Benzene, (methoxymethyl)-	Ether	fruity	-	up	-
Butane, 1,1'-oxybis[4-chloro-	Ether	-	-	up	up
Nonane, 1-iodo-	Other	-	-	-	down
3-Cyclohexene-1-carboxAldehyde, 4-(4-methyl-3-pentenyl)-	Aldehyde	citrus、floral、marine	down	-	-
(E)-2-Decenal	Aldehyde	fatty	-	up	up
Hexanal	Aldehyde	green	down	-	-
Heptanal	Aldehyde	green	-	down	-
BenzAldehyde	Aldehyde	fruity	up	up	-
BenzeneacetAldehyde	Aldehyde	green	up	-	-
(E)-2-Octenal	Aldehyde	fatty	-	up	up
2-Nonenal	Aldehyde	green、waxy、cucumber	-	-	up
4-Isopropylcyclohexa-1,3-dienecarbAldehyde	Aldehyde	fatty、spicy	-	up	up
Undecanal	Aldehyde	waxy、green	down	-	-

Compounds	Class	Odour quality	Type		
			T-GS_vs_M-GS	T-G1_vs_M-G1	T-G2_vs_M-G2
2-FurAldehyde diethyl acetal	Aldehyde	fruity、earthy、mushroom	down	-	-
BenzAldehyde, 2-methyl-	Aldehyde	cherry	up	-	-
cis-7-Decen-1-al	Aldehyde	citrus	up	up	up
BenzeneacetAldehyde, .alpha.-methyl-4-(2-methylpropyl)-	Aldehyde	-	up	-	down
Z-11-Pentadecenal	Aldehyde	-	-	down	-
2,6,6-trimethyl-1-Cyclohexene-1-acetAldehyde	Aldehyde	camphoreous	-	-	down
2-hydroxy-BenzAldehyde	Aldehyde	medicinal	up	up	up
Hexanoic Acid	Acid	fatty	-	down	-
Nonanoic Acid	Acid	waxy	-	-	down
6-Octenoic Acid, 3,7-dimethyl-	Acid	green、grassy、floral	down	-	-
8-Methyl-6-nonenoic Acid	Acid	-	-	-	up
2-Propenoic Acid, 3-phenyl-	Acid	balsamic	down	down	-
9,12,15-Octadecatrienoic Acid, (Z,Z,Z)-	Acid	fatty	down	-	-
Gamolenic Acid	Acid	-	down	-	-
n-Hexadecanoic Acid	Acid	waxy	up	up	-
1-Methylcyclohexylcarboxylic Acid	Acid	-	up	up	up
cis-3-Hexenyl cis-3-hexenoate	Acid	green	-	-	down
2-Octenoic Acid	Acid	-	up	-	-
Pentanoic Acid, 2-methyl-	Acid	cheesy	-	up	-
(1aR,1bS,2aS,5S,5aS,7aS)-2,2,5,7a-Tetramethyldecahydrocyclopenta[2',3']cyclobuta[1',2':3,4]benzo[1,2-b]oxirene	Terpenoids	-	up	down	-
Kessane	Terpenoids	-	down	-	-

Compounds	Class	Odour quality	Type		
			T-GS_vs_M-GS	T-G1_vs_M-G1	T-G2_vs_M-G2
.alpha.-Pinene	Terpenoids	herbal	down	down	-
3-Carene	Terpenoids	citrus	down	down	-
Linalool	Terpenoids	floral	down	-	-
terpinolene	Terpenoids	herbal	up	up	up
Carveol	Terpenoids	minty	up	up	-
(-)-Carvone	Terpenoids	minty	-	up	up
2-Cyclohexen-1-ol, 3-methyl-6-(1-methylethyl)-, trans-	Terpenoids	herbal	-	up	up
2,6-Octadienal, 3,7-dimethyl-, (Z)-	Terpenoids	citrus	up	-	-
4-(1-methylethyl)-Benzaldehyde	Terpenoids	spicy	down	-	-
Geraniol	Terpenoids	floral	-	up	up
safranal	Terpenoids	herbal	up	up	up
(E)-.beta.-Famesene	Terpenoids	woody	-	down	down
.alpha.-Farnesene	Terpenoids	woody	-	-	down
Naphthalene, 1,2,3,5,6,8a-hexahydro-4,7-dimethyl-1-(1-methylethyl)-, (1S-cis)-	Terpenoids	herbal	-	-	down
Cyclohexene, 3-(1,5-dimethyl-4-hexenyl)-6-methylene-, [S-(R*,S*)]-	Terpenoids	herbal、fruity、woody	down	-	-
.alpha.-Calacorene	Terpenoids	woody	-	down	-
(E)-4,8-Dimethylnona-1,3,7-triene	Terpenoids	-	up	down	down
p-Menth-8-en-1-ol, stereoisomer	Terpenoids	-	-	up	up
(-)-trans-Isopiperitenol	Terpenoids	-	-	up	up
6-Octen-1-ol, 3,7-dimethyl-, (R)-	Terpenoids	floral	-	up	up
2,6-Octadien-1-ol, 3,7-dimethyl-	Terpenoids	-	-	up	up
Bicyclo[2.2.1]heptane-2,5-dione, 1,7,7-trimethyl-	Terpenoids	-	-	down	-

Compounds	Class	Odour quality	Type		
			T-GS_vs_M-GS	T-G1_vs_M-G1	T-G2_vs_M-G2
2-Cyclohexen-1-one, 2-hydroxy-3-methyl-6-(1-methylethyl)-	Terpenoids	minty	up	-	-
Furan, 3-(4,8-dimethyl-3,7-nonadienyl)-, (E)-	Terpenoids	-	up	-	-
Pinocarvone	Terpenoids	minty	-	up	up
beta-Elemen	Terpenoids	sweet	up	-	-
(+)-alpha-Pinene	Terpenoids	herbal	down	down	-
Isopinocarveol	Terpenoids	woody	-	up	up
d-Nerolidol	Terpenoids	floral	down	-	-
Benzene, 1-(1,5-dimethylhexyl)-4-methyl-	Terpenoids	-	-	-	down
(+)-3-Carene	Terpenoids	sweet	down	down	-
1,3,6,10-Cyclotetradecatetraene, 3,7,11-trimethyl-14-(1-methylethyl)-, [S-(E,Z,E,E)]-	Terpenoids	-	up	-	-
Farnesene epoxide, E-	Terpenoids	-	-	-	up
o-Cymene	Terpenoids	-	-	-	up
(1S,4S,4aS)-1-Isopropyl-4,7-dimethyl-1,2,3,4,4a,5-hexahydronaphthalene	Terpenoids	-	-	-	down
Benzene, 1-methyl-4-(1,2,2-trimethylcyclopentyl)-, (R)-	Terpenoids	-	down	-	-
Naphthalene, 1,2,3,4,4a,7-hexahydro-1,6-dimethyl-4-(1-methylethyl)-	Terpenoids	-	down	-	-
1-Phenanthrenecarboxaldehyde, 1,2,3,4,4a,9,10,10a-octahydro-1,4a-dimethyl-7-(1-methylethyl)-, [1R-(1.alpha.,4a.beta.,10a.alpha.)]-	Terpenoids	-	down	-	-
p-Mentha-1,5,8-triene	Terpenoids	roasted	-	down	down
(-)-Aristolene	Terpenoids	-	-	down	-
Bicyclo[3.1.0]hex-2-ene, 4-methylene-1-(1-methylethyl)-	Terpenoids	-	-	up	up

Compounds	Class	Odour quality	Type		
			T-GS_vs_M-GS	T-G1_vs_M-G1	T-G2_vs_M-G2
Cyclopropanecarboxylic Acid, 2,2-dimethyl-3-(2-methyl-1-propenyl)-, (1R-trans)-	Terpenoids	-	-	-	up
1(2H)-Naphthalenone, 3,4,4a,5,6,7-hexahydro-4a,5-dimethyl-3-(1-methylethenyl)-, [3S-(3.alpha.,4a.alpha.,5.alpha.)]-	Terpenoids	-	up	-	-
Longifolenaldehyde	Terpenoids	-	up	-	-
Tricyclo[2.2.1.0(2,6)]heptane-3-methanol, 2,3-dimethyl-	Terpenoids	-	-	-	up
(1S,1aS,1bR,4S,5S,5aS,6aR)-1a,1b,4,5a-Tetramethyldecahydro-1,5-methanocyclopropa[a]indene	Terpenoids	-	-	up	up
1-Isopropyl-4,7-dimethyl-1,2,3,5,6,8a-hexahydronaphthalene	Terpenoids	-	down	down	-
Bicyclo[3.2.1]oct-2-ene, 3-methyl-4-methylene-	Terpenoids	-	up	up	up
Carvenone	Terpenoids	minty	-	up	-
1-(2,6,6-trimethyl-1-cyclohexen-1-yl)-1-Penten-3-one	Terpenoids	orris	up	-	-
(1R,5R)-2-Methyl-5-((R)-6-methylhept-5-en-2-yl)bicyclo[3.1.0]hex-2-ene	Terpenoids	-	-	down	down
Adamantane	Hydrocarbons	-	down	-	-
Nonane	Hydrocarbons	gasoline	up	-	up
Decane	Hydrocarbons	-	-	-	down
Pentadecane	Hydrocarbons	-	-	-	down
Heptadecane	Hydrocarbons	-	up	-	-
Heptane, 2,2,4,6,6-pentamethyl-	Hydrocarbons	-	down	-	down
1,7-Nonadiene, 4,8-dimethyl-	Hydrocarbons	-	up	up	up
Nonane, 3,7-dimethyl-	Hydrocarbons	-	-	-	down
Heptane, 2,4-dimethyl-	Hydrocarbons	-	-	-	up
4-methyl-1-(1-methylethyl)-Bicyclo[3.1.0]hex-2-ene	Hydrocarbons	-	down	up	down

Compounds	Class	Odour quality	Type		
			T-GS_vs_M-GS	T-G1_vs_M-G1	T-G2_vs_M-G2
2,4-Dimethyldodecane	Hydrocarbons	-	-	up	-
Dodecane, 4-methyl-	Hydrocarbons	-	up	up	-
Dodecane, 4,6-dimethyl-	Hydrocarbons	-	-	-	down
Dodecane, 2,6,11-trimethyl-	Hydrocarbons	-	-	up	-
Tetradecane, 4-methyl-	Hydrocarbons	-	up	-	-
Undecane, 2,5-dimethyl-	Hydrocarbons	-	up	up	-
Tridecane, 2-methyl-	Hydrocarbons	-	-	-	down
Heptadecane, 2-methyl-	Hydrocarbons	-	down	down	-
Decane, 5-methyl-	Hydrocarbons	-	-	up	-
Undecane, 2,4-dimethyl-	Hydrocarbons	-	-	up	up
Undecane, 2-methyl-	Hydrocarbons	-	-	up	up
1,4,7,-Cycloundecatriene, 1,5,9,9-tetramethyl-, Z,Z,Z-	Hydrocarbons	-	up	up	down
3,5-Dimethyldodecane	Hydrocarbons	-	-	down	up
Undecane, 3,4-dimethyl-	Hydrocarbons	-	-	up	up
Undecane, 3,8-dimethyl-	Hydrocarbons	-	-	-	down
Dodecane, 5-methyl-	Hydrocarbons	-	-	up	-
Pentadecane, 4-methyl-	Hydrocarbons	-	-	down	-
Undecane, 4,7-dimethyl-	Hydrocarbons	-	-	-	down
Nonane, 2,6-dimethyl-	Hydrocarbons	-	-	up	up
Nonane, 2,2,4,4,6,8,8-heptamethyl-	Hydrocarbons	-	-	down	-
1,5-Heptadiene, 2,6-dimethyl-	Hydrocarbons	-	down	-	-
Undecane, 3,5-dimethyl-	Hydrocarbons	-	-	up	up
Octane, 2,6,6-trimethyl-	Hydrocarbons	-	-	up	up
Bicyclo(3.3.1)non-2-ene	Hydrocarbons	-	-	up	up

Compounds	Class	Odour quality	Type		
			T-GS_vs_M-GS	T-G1_vs_M-G1	T-G2_vs_M-G2
Heptane, 3,3,5-trimethyl-	Hydrocarbons	-	-	-	down
Undecane, 4-methyl-	Hydrocarbons	-	-	up	up
Dodecane, 3-methyl-	Hydrocarbons	-	-	up	up
Nonane, 3-methyl-5-propyl-	Hydrocarbons	-	up	-	-
2-Methyl-7-exo-vinylbicyclo[4.2.0]oct-1(2)-ene	Hydrocarbons	-	-	down	down
3,7-dimethyl-Undecane	Hydrocarbons	-	-	-	down
octyl-Cyclohexane	Hydrocarbons	-	-	down	down
3-methyl-Pentadecane	Hydrocarbons	-	-	down	-
2-butyl-1,3,3-trimethyl-Cyclohexene	Hydrocarbons	-	-	down	-
Hexacosane	Hydrocarbons	-	down	-	-
Bicyclo[3.1.1]heptan-3-one, 2-hydroxy-2,6,6-trimethyl-	Ketone	-	-	up	up
1,2-Cyclohexanedione	Ketone	nutty	-	up	up
Cyclopentanone, 2-(2-hexenyl)-	Ketone	floral	down	-	-
4-Acetylanisole	Ketone	anisic	up	-	-
2-methyl-3-Pentanone	Ketone	mint	down	-	-
5-Hepten-2-one, 6-methyl-	Ketone	citrus	-	down	-
(5Z)-Octa-1,5-dien-3-one	Ketone	green、geranium、metallic	-	-	down
3,5-Octadien-2-one	Ketone	fatty、fruity、mushroom	down	down	-
2-Pentadecanone, 6,10,14-trimethyl-	Ketone	floral	-	up	-
Isophorone	Ketone	woody	up	-	-
1-Pentanone, 1-(p-anisyl)-3-methyl-	Ketone	-	-	-	down
Ethanone, 1-(2-methyl-1-cyclopenten-1-yl)-	Ketone	-	-	down	down

Compounds	Class	Odour quality	Type		
			T-GS_vs_M-GS	T-G1_vs_M-G1	T-G2_vs_M-G2
Cyclohexanone, 2,2,6-trimethyl-	Ketone	thujonic、honey、cistus	up	-	-
2-Acetyl-1,3-cyclohexanedione	Ketone	-	-	up	up
Ethanone, 1-(2,4-dimethylphenyl)-	Ketone	floral	up	-	-
4-Isopropyl-1,3-cyclohexanedione	Ketone	-	up	up	up
4,6-Octadiyn-3-one, 2-methyl-	Ketone	-	down	down	down
9-Cedranone	Ketone	-	-	down	-
2-Ethoxy-3-methylpyrazine	Heterocyclic compound	nutty	down	-	-
5,6,7,8-Tetrahydroquinoxaline	Heterocyclic compound	nutty	down	-	-
Sesquirosefuran	Heterocyclic compound	-	down	-	-
Pyridine	Heterocyclic compound	fishy	-	down	-
Ethanone, 1-(2-furanyl)-	Heterocyclic compound	balsamic	down	-	-
Furan, 2-pentyl-	Heterocyclic compound	fruity、green	up	-	-
Ethanone, 1-(2-pyridinyl)-	Heterocyclic compound	popcorn	up	-	-
Acetylpyrazine	Heterocyclic compound	popcorn	up	-	-
Ethanone, 1-(1H-pyrrol-2-yl)-	Heterocyclic compound	musty	up	up	up
2-Furanmethanol, 5-ethenyltetrahydro-.alpha.,.alpha.,5-trimethyl-, cis-	Heterocyclic compound	earthy、floral	-	up	-
rose furan epoxide	Heterocyclic compound	green	up	-	-
Indole	Heterocyclic compound	animal	up	-	-
4-Methylthiazole	Heterocyclic compound	nutty	-	-	up
Coumarin	Heterocyclic compound	tonka	-	-	down
8-Azabicyclo[3.2.1]octan-3-ol, 8-methyl-, endo-	Heterocyclic compound	-	up	up	up
5,6-Dihydro-5-methyluracil	Heterocyclic compound	-	up	up	-

Compounds	Class	Odour quality	Type		
			T-GS_vs_M-GS	T-G1_vs_M-G1	T-G2_vs_M-G2
Pentylenetetrazol	Heterocyclic compound	-	down	-	-
GrAmine	Heterocyclic compound	-	up	down	-
2,6-Dihydroxy-7-methylpurine	Heterocyclic compound	-	-	-	up
2-methyl-4-Quinolinol	Heterocyclic compound	-	up	-	-
[1,3]Dioxolo[4,5-j]phenanthridine	Heterocyclic compound	-	down	-	-
Nifedipine	Heterocyclic compound	-	down	-	-
2(5H)-Furanone	Heterocyclic compound	buttery	-	-	up
1H-Pyrrole-3-carbonitrile	Heterocyclic compound	-	up	-	-
1,4 Benzodioxan-6-amine	Heterocyclic compound	-	-	down	-
2-Ethylpiperidine	Heterocyclic compound	-	-	up	up
Piperidin-3-one, 2,2,5-methyl-	Heterocyclic compound	-	up	-	-
2H-Pyran-2-methanol, tetrahydro-	Heterocyclic compound	-	down	-	down
1-Pentanone, 1-(2-furanyl)-	Heterocyclic compound	sweet、caramellic	up	-	-
2H-Pyran-2-one, tetrahydro-4,6,6-trimethyl-	Heterocyclic compound	-	up	up	up
6-Quinolinamine, 2-methyl-	Heterocyclic compound	-	-	-	up
2-Piperidinimine	Heterocyclic compound	-	-	-	up
Furan, 2-(1-pentenyl)-, (E)-	Heterocyclic compound	roasted	down	-	down
1,2,4,5-Tetrazin-3-Amine	Heterocyclic compound	-	-	up	up
1,2,4,5-Tetrazine-3,6-diAmine	Heterocyclic compound	-	up	-	-
3-Methyl-4,5-diamino-1,2,4(4H)-triazole	Heterocyclic compound	-	-	up	up
4H-Pyran-4-one, 2,3-dihydro-3,5-dihydroxy-6-methyl-	Heterocyclic compound	-	up	-	up
3-Methylbenzothiophene	Heterocyclic compound	-	down	-	-
1,7-Diazabicyclo[2.2.0]heptane	Heterocyclic compound	-	up	-	-
2-Thiophenecarboxaldehyde, 5-chloro-	Heterocyclic compound	-	up	-	up

Compounds	Class	Odour quality	Type		
			T-GS_vs_M-GS	T-G1_vs_M-G1	T-G2_vs_M-G2
2-Hydroxymethylbenzimidazole	Heterocyclic compound	-	-	-	down
1-Pentanone, 1-(2-thienyl)-	Heterocyclic compound	-	down	-	-
2,5-Furandione, 3-methyl-4-propyl-	Heterocyclic compound	-	down	-	-
5-Isopropyl-3,3-dimethyl-2-methylene-2,3-dihydrofuran	Heterocyclic compound	-	up	-	-
2,5-Furandione, dihydro-3-methyl-	Heterocyclic compound	-	-	up	up
1H-Tetrazole	Heterocyclic compound	-	up	up	up
2-Furancarboxylic acid, tetrahydro-3-methyl-5-oxo-	Heterocyclic compound	-	-	up	up
1H-Imidazole-4-methanol	Heterocyclic compound	-	down	-	-
8-Azabicyclo[3.2.1]octane, 3-chloro-8-methyl-	Heterocyclic compound	-	-	up	-
2,6-Piperidinedione, 3-ethyl-	Heterocyclic compound	-	-	up	up
N-Benzyloxy-2-carbomethoxyaziridine	Heterocyclic compound	-	up	-	-
3-FurAldehyde	Heterocyclic compound	-	up	up	up
(2R,4aS,5S,8aS)-2,5-Dipentyldecahydroquinoline	Heterocyclic compound	-	down	-	-
diethylboryl-.delta.-Valerolactam	Heterocyclic compound	-	up	-	-
2-Thiopheneacetic acid, but-3-yn-2-yl ester	Heterocyclic compound	-	-	down	-
6-methyl-3(2H)-Pyridazinone	Heterocyclic compound	-	down	down	-
Hexanoic acid, 3-methyl-2-butenyl ester	Ester	fruity	-	-	up
Propanoic acid, 2,2-dimethyl-, pentyl ester	Ester	-	-	up	up
Butanoic acid, 2-methyl-, 2-methylbutyl ester	Ester	fruity	-	-	up
n-Capric acid isobutyl ester	Ester	fermented、oily、sweet	down	-	-
1-Hexanol, 5-methyl-2-(1-methylethyl)-, acetate	Ester	-	-	up	up
Butanoic acid, 3-methyl-, hexyl ester	Ester	fruity	-	up	up
Acetic acid, hexyl ester	Ester	fruity	-	up	up

Compounds	Class	Odour quality	Type		
			T-GS_vs_M-GS	T-G1_vs_M-G1	T-G2_vs_M-G2
3-Mercapto-3-methylbutyl formate (ester)	Ester	sulfurous	up	up	up
Butanoic acid, 3-hexenyl ester, (Z)-	Ester	green	down	down	down
cis-3-Hexenyl-.alpha.-methylbutyrate	Ester	green	-	down	down
1,6-Octadien-3-ol, 3,7-dimethyl-, formate	Ester	herbal	-	-	up
trans-Geranic acid methyl ester	Ester	waxy	down	-	-
Acetic acid, nonyl ester	Ester	waxy	down	-	-
trans-3-Methyl-4-octanolide	Ester	coconut	-	up	-
Hexanoic acid, 3-hexenyl ester, (Z)-	Ester	green	-	-	down
Hexanoic acid, hexyl ester	Ester	green	-	-	down
Butanoic acid, 3-methyl-, 1-ethenyl-1,5-dimethyl-4-hexenyl ester	Ester	herbal、cirtus	up	-	-
Butanoic acid, 3,7-dimethyl-2,6-octadienyl ester, (E)-	Ester	fruity	-	-	down
Benzoic acid, 1-methylethyl ester	Ester	floral	up	-	-
Hexanedioic acid, dimethyl ester	Ester	nutty	-	-	up
2-Ethylhexyl acrylate	Ester	-	-	up	up
Fenobucarb	Ester	-	up	-	-
n-Caproic acid vinyl ester	Ester	-	-	-	up
2-Oxepanone	Ester	-	-	up	up
E-2-Hexenyl benzoate	Ester	-	down	-	-
Butanoic Acid, 3-methylbutyl ester	Ester	fruity	-	up	up
Methylparaben	Ester	-	-	down	-
4-Decenoic acid, methyl ester, Z-	Ester	fruity、fishy、green	down	-	-
9,12,15-Octadecatrienoic acid, methyl ester	Ester	oily、fatty、fruity	down	-	-
Fenchyl acetate	Ester	balsamic	up	up	up
Isopropyl myristate	Ester	fatty	up	up	up

Compounds	Class	Odour quality	Type		
			T-GS_vs_M-GS	T-G1_vs_M-G1	T-G2_vs_M-G2
n-Propyl acetate	Ester	fruity	-	-	down
3-Hexen-1-ol benzoate	Ester	-	-	-	down
9,12,15-Octadecatrienoic acid, methyl ester, (Z,Z,Z)-	Ester	-	-	up	-
Sulfurous acid, isobutyl pentyl ester	Ester	-	-	up	-
Benzoic acid, 2-propenyl ester	Ester	fruity	up	-	-
l-Alanine, N-(2,3,4-trifluorobenzoyl)-, methyl ester	Ester	-	-	-	down
4-Hexen-1-ol, 5-methyl-2-(1-methylethenyl)-, acetate	Ester	-	-	up	up
(E)-Hex-3-enyl (E)-2-methylbut-2-enoate	Ester	-	-	down	-
4-Methylhexyl 3-methylbutanoate	Ester	-	down	-	-
Butanoic acid, cyclopentyl ester	Ester	-	down	-	-
Butanoic acid, 1-methylhexyl ester	Ester	fruity	-	up	up
Ethyl mandelate	Ester	-	up	-	-
Propanoic acid, 2-methyl-, butyl ester	Ester	fruity	up	-	-
Propanoic acid, 2-methyl-, 2-methylbutyl ester	Ester	fruity	-	up	up
Acetic acid, (propylthio)-, methyl ester	Ester	-	up	-	-
Hexanoic acid, 2-methylbutyl ester	Ester	ethereal	-	up	up
Hexanoic acid, pentyl ester	Ester	fruity	-	up	up
Propanoic acid, 2-methyl-, propyl ester	Ester	fruity	-	-	up
1-Octyl trifluoroacetate	Ester	-	-	up	up
Benzoic acid, 3,4-dimethyl-, methyl ester	Ester	-	up	-	up
Octanoic acid, 3-hexenyl ester, (Z)-	Ester	green	-	-	down
Pentadecanoic acid, 14-methyl-, methyl ester	Ester	-	down	-	-
(6,6-dimethyl-2-bicyclo[3.1.1]hept-2-enyl)methyl 2-methylpropanoate	Ester	woody、fruity、pine	-	down	-

Compounds	Class	Odour quality	Type		
			T-GS_vs_M-GS	T-G1_vs_M-G1	T-G2_vs_M-G2
Valeric acid, 4-nitrophenyl ester	Ester	-	up	-	-
Sulfurous acid, hexyl pentadecyl ester	Ester	-	-	-	down
Sulfurous acid, 2-ethylhexyl hexyl ester	Ester	-	-	-	down
Pentadecafluorooctanoic acid, 2-ethylhexyl ester	Ester	-	-	-	down
2-(2-butoxyethoxy)-Ethanol,acetate	Ester	-	-	down	-
(Z,Z)-9,15-Octadecadienoic acid, methyl ester	Ester	-	up	-	-
(E)-Hexanoic acid, 2-hexenyl ester	Ester	green	-	-	down

Table S4. The 53 volatile components with higher content in M-GS.

No.	Compounds	Class	Odour quality
1	CyclohexanAmine, N-cyclohexyl-	Amine	-
2	3,7-Octadiene-2,6-diol, 2,6-dimethyl-	Alcohol	-
3	Cyclobutaneethanol, 1-methyl-2-(1-methylethenyl)-, cis-	Alcohol	-
4	4-Hexen-1-ol, acetate	Alcohol	-
5	3,6-Nonadien-1-ol, (E,Z)-	Alcohol	green
6	3-Hexen-1-ol, (Z)-	Alcohol	green
7	(5-bromopentyl)-Benzene,	Aromatics	-
8	Benzene, (2-nitroethyl)-	Aromatics	flower、spice
9	Naphthalene, 1,2,3,4,4a,5,6,7-octahydro-4a-methyl-	Aromatics	-
10	Benzene, 1,4-dichloro-	Aromatics	-
11	Benzene, 2-ethyl-1,4-dimethyl-	Aromatics	-
12	p-Xylene	Aromatics	plastic, green, pungent
13	Phenol, 4-propyl-	Phenol	phenolic、medicinal
14	1-iodo-Decane	Halogenated hydrocarbons	-
15	BenzAldehyde, 2-methyl-	Aldehyde	cherry
16	BenzeneacetAldehyde	Aldehyde	green
17	2-Octenoic Acid	Acid	-
18	1,3,6,10-Cyclotetradecatetraene, 3,7,11-trimethyl-14-(1-methylethyl)-, [S-(E,Z,E,E)]-	Terpenoids	-
19	(1aR,1bS,2aS,5S,5aS,7aS)-2,2,5,7a-Tetramethyldecahydrocyclopenta[2',3']cyclobuta[1',2':3,4]benzo[1,2-b]oxirene	Terpenoids	-
20	Longifolenaldehyde	Terpenoids	-
21	Furan, 3-(4,8-dimethyl-3,7-nonadienyl)-, (E)-	Terpenoids	-
22	1(2H)-Naphthalenone, 3,4,4a,5,6,7-hexahydro-4a,5-dimethyl-3-(1-methylethenyl)-, [3S-(3.alpha.,4a.alpha.,5.alpha.)]-	Terpenoids	-
23	beta-Elemen	Terpenoids	sweet
24	2-Cyclohexen-1-one, 2-hydroxy-3-methyl-6-(1-methylethyl)-	Terpenoids	minty
25	2,6-Octadienal, 3,7-dimethyl-, (Z)-	Terpenoids	citrus
26	(E)-4,8-Dimethylnona-1,3,7-triene	Terpenoids	-
27	safranal	Terpenoids	herbal
28	Nonane, 3-methyl-5-propyl-	Hydrocarbons	-
29	Nonane	Hydrocarbons	gasoline
30	4-Acetylanisole	Ketone	anisic
31	Ethanone, 1-(2,4-dimethylphenyl)-	Ketone	floral
32	Cyclohexanone, 2,2,6-trimethyl-	Ketone	thujonic、pungent
33	Isophorone	Ketone	woody
34	diethylboryl-.delta.-Valerolactam	Heterocyclic compound	-
35	N-Benzyloxy-2-carbomethoxyaziridine	Heterocyclic compound	-

No.	Compounds	Class	Odour quality
36	GrAmine	Heterocyclic compound	-
37	rose furan epoxide	Heterocyclic compound	green
38	2-methyl-4-Quinolinol	Heterocyclic compound	-
39	5-Isopropyl-3,3-dimethyl-2-methylene-2,3-dihydrofuran	Heterocyclic compound	-
40	1-Pentanone, 1-(2-furanyl)-	Heterocyclic compound	sweet、caramellic
41	Piperidin-3-one, 2,2,5-methyl-	Heterocyclic compound	-
42	Furan, 2-pentyl-	Heterocyclic compound	fruity、green、earthy
43	5,6-Dihydro-5-methyluracil	Heterocyclic compound	-
44	1,2,4,5-Tetrazine-3,6-diAmine	Heterocyclic compound	-
45	1,7-Diazabicyclo[2.2.0]heptane	Heterocyclic compound	-
46	1H-Pyrrole-3-carbonitrile	Heterocyclic compound	-
47	Fenobucarb	Ester	-
48	Butanoic acid, 3-methyl-, 1-ethenyl-1,5-dimethyl-4-hexenyl ester	Ester	herbal
49	Ethyl mandelate	Ester	-
50	Benzoic acid, 3,4-dimethyl-, methyl ester	Ester	-
51	Benzoic acid, 1-methylethyl ester	Ester	floral
52	Benzoic acid, 2-propenyl ester	Ester	fruity
53	Propanoic acid, 2-methyl-, butyl ester	Ester	fruity
